# Spontaneous low frequency BOLD signal variations from resting-state fMRI are decreased in Alzheimer disease

**DOI:** 10.1371/journal.pone.0178529

**Published:** 2017-06-05

**Authors:** Samaneh Kazemifar, Kathryn Y. Manning, Nagalingam Rajakumar, Francisco A. Gómez, Andrea Soddu, Michael J. Borrie, Ravi S. Menon, Robert Bartha, for the Alzheimer’s Disease Neuroimaging Initiative

**Affiliations:** 1 Centre for Functional and Metabolic Mapping, Robarts Research Institute, University of Western Ontario, London, Ontario, Canada; 2 Department of Medical Biophysics, University of Western Ontario, London, Ontario, Canada; 3 Department of Anatomy and Cell Biology, University of Western Ontario, London, Ontario, Canada; 4 Department of Mathematics, Universidad Nacional de Colombia, Sede Bogotá, Colombia; 5 Department of Physics and Astronomy, University of Western Ontario, London, Ontario, Canada; 6 Department of Medicine, University of Western Ontario, London, Ontario, Canada; 7 Division of Aging, Rehabilitation and Geriatric Care, Lawson Health Research Institute, London, Ontario, Canada; Banner Alzheimer's Institute, UNITED STATES

## Abstract

Previous studies have demonstrated altered brain activity in Alzheimer's disease using task based functional MRI (fMRI), network based resting-state fMRI, and glucose metabolism from ^18^F fluorodeoxyglucose-PET (FDG-PET). Our goal was to define a novel indicator of neuronal activity based on a first-order textural feature of the resting state functional MRI (RS-fMRI) signal. Furthermore, we examined the association between this neuronal activity metric and glucose metabolism from ^18^F FDG-PET. We studied 15 normal elderly controls (NEC) and 15 probable Alzheimer disease (AD) subjects from the AD Neuroimaging Initiative. An independent component analysis was applied to the RS-fMRI, followed by template matching to identify neuronal components (NC). A regional brain activity measurement was constructed based on the variation of the RS-fMRI signal of these NC. The standardized glucose uptake values of several brain regions relative to the cerebellum (SUVR) were measured from partial volume corrected FDG-PET images. Comparing the AD and NEC groups, the mean brain activity metric was significantly lower in the accumbens, while the glucose SUVR was significantly lower in the amygdala and hippocampus. The RS-fMRI brain activity metric was positively correlated with cognitive measures and amyloid β_1–42_ cerebral spinal fluid levels; however, these did not remain significant following Bonferroni correction. There was a significant linear correlation between the brain activity metric and the glucose SUVR measurements. This proof of concept study demonstrates that this novel and easy to implement RS-fMRI brain activity metric can differentiate a group of healthy elderly controls from a group of people with AD.

## Introduction

Alzheimer disease is considered to be a progressive neurodegenerative condition clinically characterized by cognitive dysfunction and memory impairments [1] that appear to result from the pathological accumulation of amyloid plaques and neurofibrillary tangles [2, 3]. Brain atrophy measured by magnetic resonance imaging (MRI) has been established as an important biomarker associated with disease progression and treatment response [4–6]. Another established biomarker is reduced regional uptake of ^18^F labeled fluorodeoxyglucose (FDG) measured using positron emission tomography (PET) [7], indicating lower glucose metabolism in Alzheimer’s disease [8, 9].

Neuronal activity can also be inferred from blood oxygen level dependent (BOLD) contrast as exploited in functional MRI (fMRI). More recently, resting state fMRI (RS-fMRI) measures of spontaneous low frequency fluctuations (< 0.1 HZ) in the BOLD signal have been used to identify functionally connected brain regions (networks) without the performance of an overt task [10, 11]. Previous studies [12, 13] have shown that several resting state networks can be identified, and may be altered in Alzheimer’s disease. For example, multiple studies have found RS-fMRI can be used to show disrupted connectivity between the hippocampus and other brain regions in Alzheimer disease [13–15]. Furthermore, the interconnectivity of brain regions can be used to classify subjects with Alzheimer disease, amnestic mild cognitive impairment, and healthy elderly [16].

A number of different statistical and mathematic approaches have been used to infer functional connectivity from RS-fMRI data. One common approach is to use the *a-priori* selection of a seed region of interest (ROI) [17–19] to determine the correlation between the mean BOLD signal time course within the ROI and the BOLD signal time courses of all other pixels in the brain. However the requirement for *a-priori* seed selection makes it difficult to examine the functional connectivity across the whole brain. Another popular multivariate technique for analyzing whole brain connectivity is independent component analysis (ICA) [20–22]. This approach does not require *a-priori* information and decomposes the BOLD signal into a set of spatial and temporal components that are maximally statistically independent [23]. The ICA method is also an efficient approach to extract scanner noise, as well as physiological and motion artifacts from the BOLD signal [24]. One of the major challenges with the ICA technique is to determine which components represent physiologically relevant networks, and which components represent noise. However methods now exist to differentiate neuronal from non-neuronal components [25].

Brain regions associated with the default mode network (DMN) have been repeatedly implicated in the pathogenesis of Alzheimer’s disease [13, 26]. FDG-PET studies have shown reduced glucose metabolism in the medial temporal cortex, hippocampus, and posterior cingulate cortex [27–29]. Decreased functional connectivity in the DMN has also been associated with increased amyloid deposition measured using Pittsburgh compound B PET [30, 31]. In addition, several studies in healthy controls have shown a relationship between glucose metabolism measured by PET and the RS-fMRI signal. For example, Di *et*. *al*. showed that metabolic activity was correlated with independent component (IC) maps of the BOLD signal in regions that are functionally connected [32]. Similarly, Tomasi *et*. *al*. [33] measured the amplitude of the RS-fMRI signal and glucose metabolism by FDG-PET and demonstrated that higher metabolism was correlated with a higher amplitude of the RS-fMRI signal in the cerebellum, occipital, and parietal cortices. Finally, Riedl *et*. *al*. [34] found a correlation between local brain activity in specific regions of interest measured from FDG-PET data and functional connectivity measured by RS-fMRI using seed-based methods.

Previous RS-fMRI studies have shown a reduction in functional connectivity between structures based on the *strength* of the correlation in the BOLD signal [18, 35]. Here, we define a novel metric of brain activity based on a first-order texture feature defined as the standard deviation of the *magnitude* of the BOLD fluctuation. The purpose of this study was to determine whether this new metric could be used to accurately differentiate healthy elderly individuals from people with mild Alzheimer disease. Our hypothesis was that the fluctuation of the magnitude of the BOLD signal as a function of time is related to neuronal activity and therefore will also correlate with FDG-PET measures of glucose metabolism. Our goal was to demonstrate the efficacy of a new indicator of Alzheimer’s disease based on the fluctuation of the magnitude of the RS-fMRI signal. Furthermore, we examined the association between the fluctuation magnitude of the neuronal derived RS-fMRI signal and FDG-PET.

## Theory

**Fig 1:**
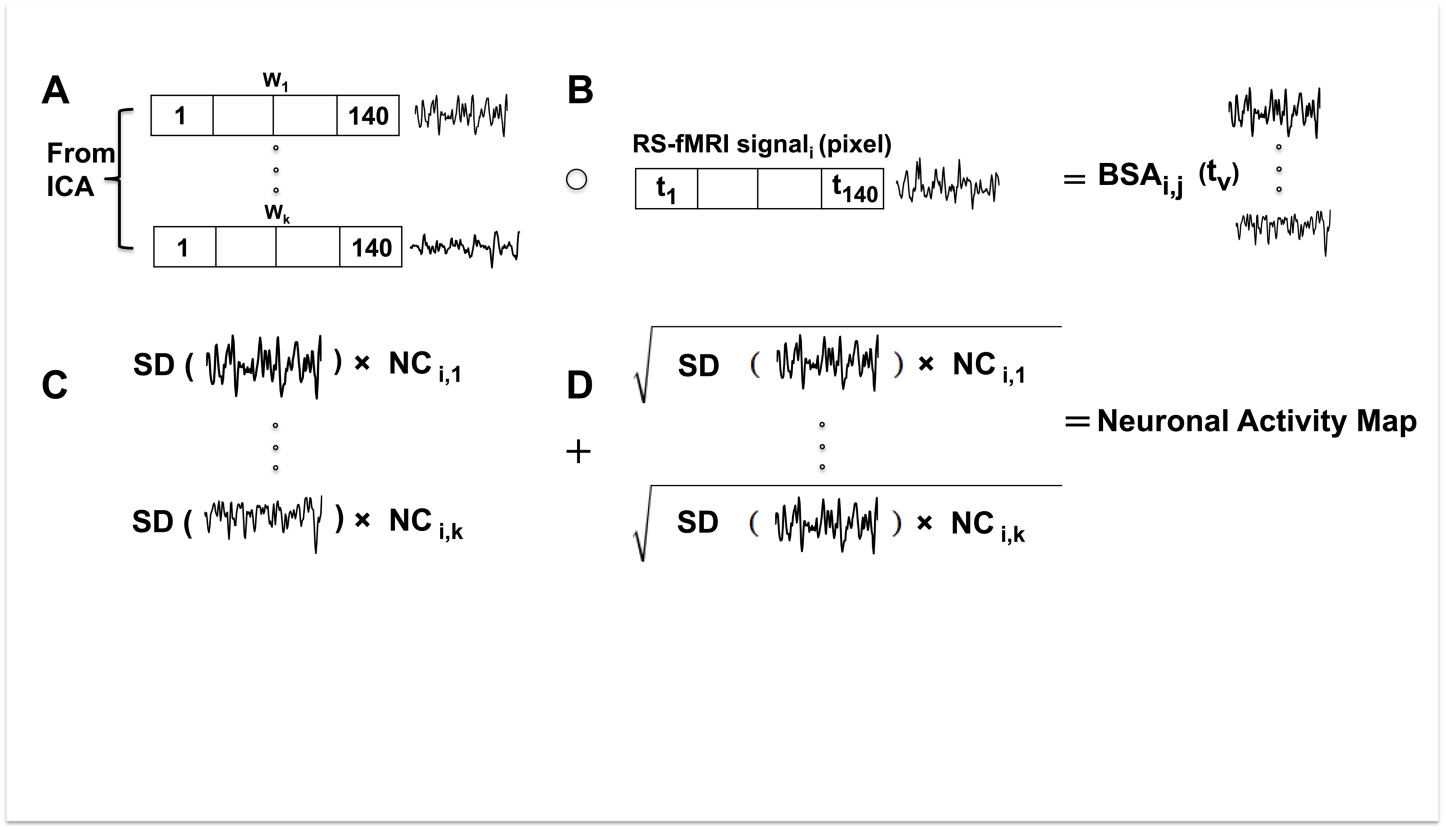
Schematic diagram of the brain activity metric calculated from the RS-fMRI signal in one pixel. The signal shown is a graphical representation of the vector data. (A) Neuronal time courses (*W*) are defined by the ICA and verified using a goodness of fit calculation, multiple template matching method and a support vector machine classifier. (B) The Hadamard product of each neuronal time course is taken with the RS-fMRI signal. (C) The standard deviation of the resulting signal is multiplied by the neuronal component. (D) The square root of the contribution of each neuronal time course is added to create a measure of neuronal activity.

In this study, we propose a brain activity measurement derived directly from the RS-fMRI signal. The RS-fMRI signal can be represented as an *i* x *v* matrix, where *i* represents each pixel, and *v* represents the number of volumes acquired in the fMRI acquisition. The ICA method decomposes the RS-fMRI signal into: 1) an *i* x *n* matrix of independent component spatial maps, where *n* represents the number of components, and 2) an *n* x *v* matrix of mixing weights (*W*) or IC time-courses. Here, the number (*k*) of legitimate ICs was identified using a goodness-of-fit (GoF) calculation, a multiple template matching method and a support vector machine (SVM) classifier [25] that included only the neuronal components (*NC*) (Fig 1A). The weighting function for each neuronal component, *W*_*k*_*(t*_*v*_*)* is then multiplied (Hadamard product, Eq 1, Fig 1B) with the equal length vector given by the RS-fMRI time series for each pixel, *S*_*i*_*(t*_*v*_*)*, generating a new vector of equal length *BSA*_*i*,*k*_(*t*_*v*_):

$$BSA_{i,k}\left(t_{v}\right)=\;S_{i}\left(t_{v}\right)\;\circ \;W_{k}\left(t_{v}\right)$$ (1)

where *BSA*_*i*,*k*_ represents the BOLD signal *amplitude* (*BSA*) for each neuronal component *k* in pixel *i*. Then, the standard deviation (SD) is calculated for *BSA*_*i*,*k*_ and is multiplied with the neuronal component (*NC*_*i*,*k*_) (Fig 1C). The sum of this metric (Eq 2, Fig 1D) for all neuronal components in a pixel represents the neuronal activity and is used to produce a neuronal activity map.

$$Neuronal\;Activity_{i}=\Sigma_{j=1}^{k}\sqrt{SD\left(BSA_{i,j}\left(t_{v}\right)\right)\;\times \;NC_{i,j}}$$ (2)

## Materials and methods

### Study subjects

Data used in the preparation of this article (S1 and S2 Tables) were obtained from the Alzheimer’s Disease Neuroimaging Initiative (ADNI) database (adni.loni.usc.edu). The ADNI was launched in 2003 as a public-private partnership, led by Principal Investigator Michael W. Weiner, MD. The primary goal of ADNI has been to test whether serial magnetic resonance imaging, positron emission tomography, other biological markers, and clinical and neuropsychological assessment can be combined to measure the progression of mild cognitive impairment and early Alzheimer’s disease. Written informed consent for participation in the study was obtained from each participant, or their family. Data acquisition was approved by the local ethics review board at each participating site.

To test the algorithm, this study included data from 15 normal elderly controls (NEC) and 15 subjects with probable Alzheimer disease of mild severity obtained from the ADNI database. We included participants that had 3.0 Tesla T_1_-weighted anatomical scans and 10-minute RS-fMRI data acquisitions. A subset of this group (13 NEC, and 11 subjects with mild Alzheimer disease) also had an FDG-PET scan available. Structural T_1_-weighted images were obtained using a sagittal 3D magnetization-prepared rapid acquisition with a gradient echo MP-RAGE sequence with pixel size 1mm×1mm×1.2mm; flip angle ~9°; TE ~4 ms; TR ~7 ms; matrix, 256x256; 170 slices [36]. The RS-fMRI scans were acquired using a single shot echo planer imaging (EPI) pulse sequence with pixel size 3.3mm×3.3mm×3.3mm; flip angle 80.0°; TE 30 ms; TR ~3000 ms; matrix = 64x64; 48 slices [37]. FDG-PET images were acquired using similar protocols on all PET scanners but varied somewhat in resolution, spacing, and dimension. However images were normalized and motion corrected [38] prior to analysis. The FDG-PET scans consisted of six-5 minute frames acquired starting 30 minutes after FDG injection [38]. All frames were registered to the first frame and then averaged to produce a single static image. All subjects were also evaluated with the mini mental state examination (MMSE) [39] to assess cognition. In addition, CSF biomarkers including total Tau protein (Tau), phosphorylated Tau protein (P-Tau) and amyloid-β (Aβ_1–42_) were obtained for all subjects (https://ida.loni.usc.edu/).

### RS-fMRI analysis

The brain was extracted from the RS-fMRI data and the anatomical T_1_-weighted images using the brain extraction tool (BET) in the FSL software (functional MRI of the brain (FMRIB) Software Library, Department of Clinical Neurosciences, University of Oxford, UK, http://fsl.fmrib.ox.ac.uk/fsl/fslwiki/FSL). The brain extracted fMRI data were preprocessed, aligned and co-registered to the MNI-152 space using the fMRI expert analysis tool (FEAT) in FSL. An initial smoothing (full-width half-maximum of 6 mm), and band-pass temporal filtering (0.01 Hz—0.1 Hz) were applied before ICA decomposition. The ICA method (30 components) was performed using the group ICA of the fMRI toolbox (GIFT) [40]. The ICA was followed by a previously described three-step process [25] to identify neuronal components in each RS-fMRI data set, all implemented in-house by Demertzi and *et*. *al*. [25]. First, a goodness of fit (GoF) calculation was used to measure the similarity value between a component and established resting state networks [25]. Second, the multiple templates matching method was applied. Then, a template had to be assigned to one of the 30 components [25]. Thirdly, a support vector machine was used to label each component as neuronal, non-neuronal, or undefined [25].

**Fig 2:**
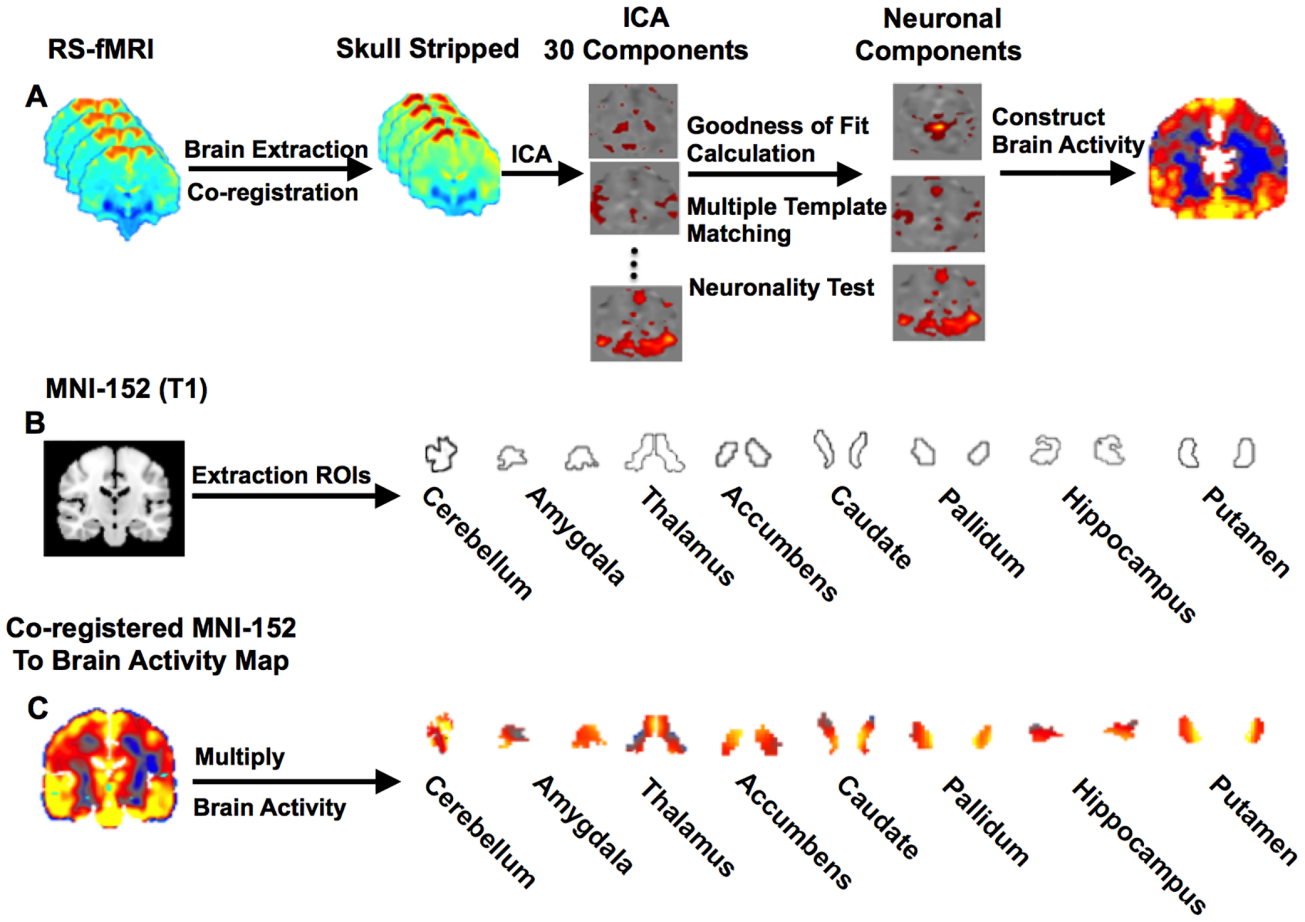
Brain activity from RS-fMRI. Schematic diagram of the brain activity (BOLD signal amplitude) measurement in different brain regions. (A) Steps involved in calculating the brain activity maps from the RS-fMRI. (B) Regions of interest extracted from MNI-152 space. (C) Activity maps within each region of interest from one subject.

Following identification of the neuronal components, a brain activity map was constructed from these components as described in the Theory section and co-registered to the MNI-152 standard image using linear and non-linear registration methods. Co-registration of the brain activity map to the anatomical image was conducted using Statistical Parametric Mapping (SPM8, Wellcome Department of Neurology, London, UK; www.fil.ion.ucl.ac.uk/spm). ROIs were defined in the MNI-152 image using the structural segmentation method called FMRIB’s integrated registration and segmentation tool (FIRST) in FSL because it is easily accessible to the research community and automated. Finally, the mean intensity from each ROI in the brain activity map was measured. A schematic representation of the steps involved in making the brain activity measurements is provided in Fig 2.

### Volumetric analysis

The FSL software was used to measure the volumes of the accumbens, amygdala, hippocampus, caudate nucleus, thalamus, pallidum, putamen, and whole brain from the T_1_-weighted image of each subject using a method similar to that previously described [41]. The volumes were normalized to the whole brain volume in each subject.

### FDG-PET analysis

The brain was also extracted from the FDG-PET images using the BET tool in FSL. A partial volume correction method was applied to each data set using the partial volume correction structural functional synergistic resolution recovery (PVC_SFSRR) software [42]. This software performs pre-processing steps including co-registration of the FDG-PET with the structural image, segmentation and smoothing (8 mm), and finally partial volume correction. The corrected FDG-PET image was co-registered to the MNI-152 template. The predefined ROIs in the MNI-152 image were applied to the co-registered FDG-PET image. The standardized uptake value (SUV) of each ROI was then normalized to the mean SUV of the cerebellum to obtain the SUV ratio (SUVR) as this has been previously shown [43] to be unchanged in healthy subjects and at the early stages of Alzheimer disease.

### Statistical analysis

Prism GraphPad (Prism, version 6.00; GraphPad Software, San Diego, CA) and Matlab toolbox (version R2010a) were used for the statistical analyses. One-way ANOVA was used to compare brain activity between the NEC and Alzheimer disease groups (*p*< 0.05 considered significant). If a significant group effect was observed, the *p-*values associated with follow-up group comparisons between brain regions were Bonferroni corrected for multiple comparisons. The association between the brain activity measured from the RS-fMRI, MMSE score, and CSF biomarkers were evaluated using linear regression. The significance of these associations was Bonferroni corrected for multiple comparisons. The Pearson correlation coefficient was used to measure the association between the brain activity in gray matter obtained from RS-fMRI and the glucose uptake from FDG-PET (*p* < 0.05 considered significant).

### Classification model

To determine if the RS-fMRI derived brain activity measurement was a suitable biomarker to differentiate patients with mild Alzheimer disease and healthy elderly controls, a SVM classifier was used. Specifically, a linear SVM [44] was trained and tested on the feature space. Regions of interest were selected based on the observed differences between groups for either the brain activity metric or FDG-PET measured glucose metabolism. A leave-one-out-test was used as a cross-validation to predict the label for each test subject (not involved in the training phase). The accuracy, sensitivity, and specificity were determined after each subject was assessed as the test subject. In the first analysis, RS-fMRI brain activity in the hippocampus and accumbens, FDG-PET in the hippocampus, and hippocampal volume were evaluated separately. In a second analysis, FDG-PET and RS-fMRI modalities were evaluated independently incorporating data from both the hippocampus and amygdala. In a third analysis, we combined the volumetric and RS-fMRI information from the hippocampus and accumbens.

## Results

### Brain maps of neuronal activity and glucose metabolism

**Fig 3:**
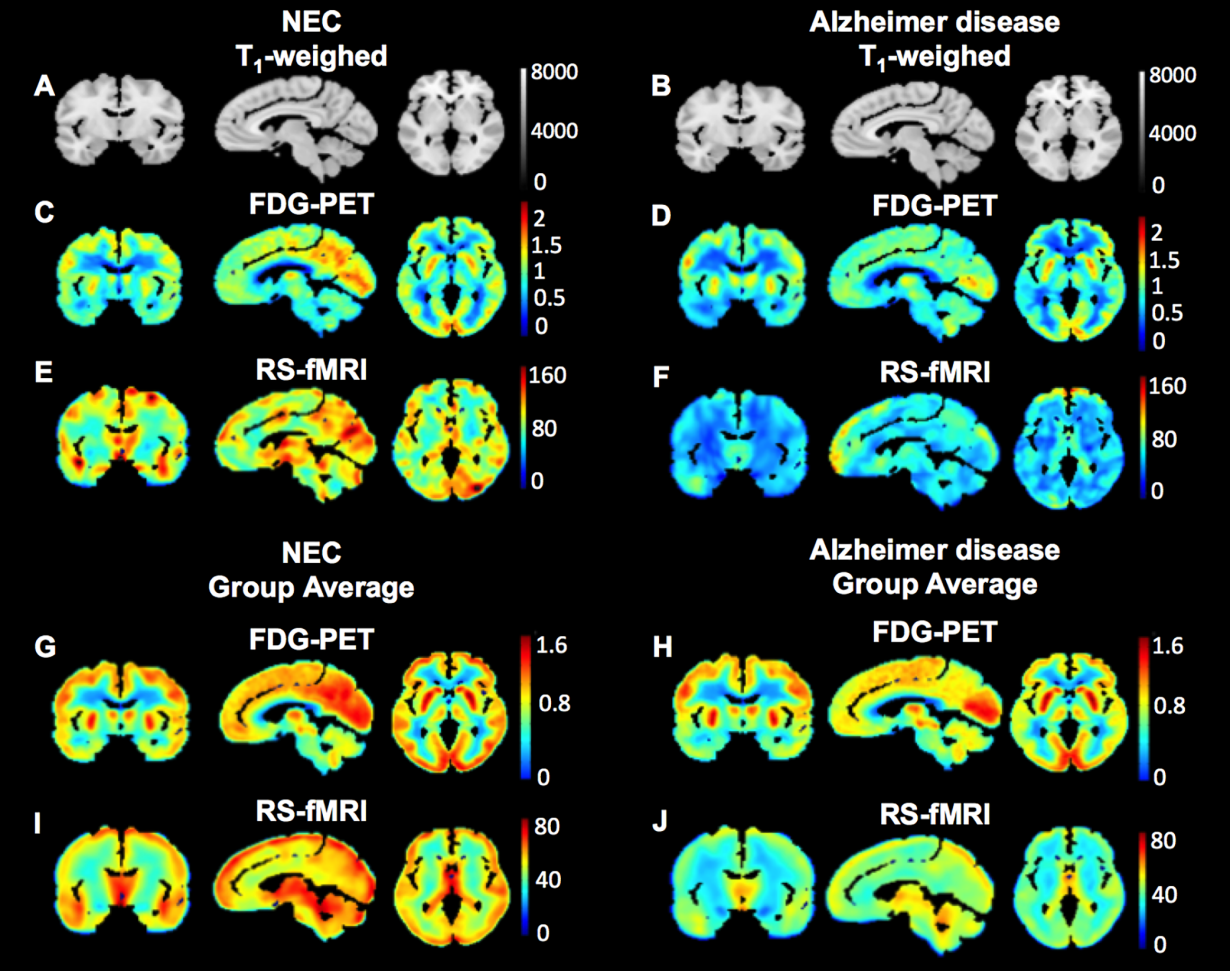
Brain activity and glucose metabolism maps. MNI-152 T_1_-weighted anatomical images in coronal, sagittal, and axial orientations (A, B). Corresponding FDG-PET SUVR images of glucose metabolism from a healthy elderly subject, and healthy elderly group averaged (C, G); a patient with Alzheimer disease, and Alzheimer disease group averaged (D, H). Brain activity maps obtained using RS-fMRI in the same healthy elderly subject, and healthy elderly group averaged (E, I) and patient with Alzheimer disease, and Alzheimer disease group averaged (F, J).

Demographic characteristics are provided in Table 1 for all 30 subjects (S1 and S2 Tables). There was no difference between group mean ages (healthy elderly subjects age range: 65–80 years; subjects with Alzheimer disease age range: 63–79 years), however as expected, there was a significant difference in the MMSE score (*p*< 0.0001, two-tailed t-test) between the two groups. Montreal neurological institute-152 (MNI-152) template T_1_-weighted images (Fig 3A and 3B) in coronal, sagittal, and axial planes are shown with corresponding standardized uptake value ratio (SUVR) glucose metabolism maps from the FDG-PET scan in one healthy elderly subject (Fig 3C) and the healthy elderly group average (Fig 3G); as well as one subject with Alzheimer disease (Fig 3D) and the Alzheimer disease group average (Fig 3H). Corresponding brain activity maps measured from the RS-fMRI in the same subjects (Fig 3E and 3F) and group averages (Fig 3I and 3J) are also provided. There is a visible decrease in signal in the subject with Alzheimer disease throughout the brain measured by both modalities.

**Table 1:** Demographic information for study participants.

	RS-fMRI NEC	RS-fMRI Alzheimer disease	FDG-PET NEC	FDG-PET Alzheimer disease
Number of subject (N)	15	15	13	11
Age (years) (Mean±SD)	73.5 ± 6.2	73.3 ±8.0	74.4 ± 6.1	70.9 ± 7.2
MMSE (Mean±SD)	28.9 ± 1.2	21.6 ± 2.1*	29.0 ± 1.1	21.8± 2.3*
Sex (F)	10	10	9	7
Tau (pg/ml) (Mean±SEM)	66.3± 9.8	164.5±22.8**	63.8±9.6	182.5±28.2**
P-Tau (pg/ml) (Mean±SEM)	38.9 ± 10.0	62.0±7.7	40.7±11.1	68.1±9.5
Aβ_1–42_ (pg/ml) (Mean±SEM)	192.5± 16.1	132.3±5.4**	179.0±15.0	131.2±7.3**

* *p*< 0.05 (two-tailed) and

** adjusted *p* value (Bonferroni) between NEC and Alzheimer disease within a single imaging modality

F = female; SEM = standard error of mean, SD = standard deviation, NEC = normal elderly controls, FDG-PET = ^18^fludeoxyglucose-PET, RS-fMRI = resting state functional MRI, MMSE = mini mental state examination, P-Tau = phospho-Tau, Aβ_1–42_ = amyloid beta_1-42_.

### Comparison of regional brain activity and glucose metabolism

**Fig 4:**
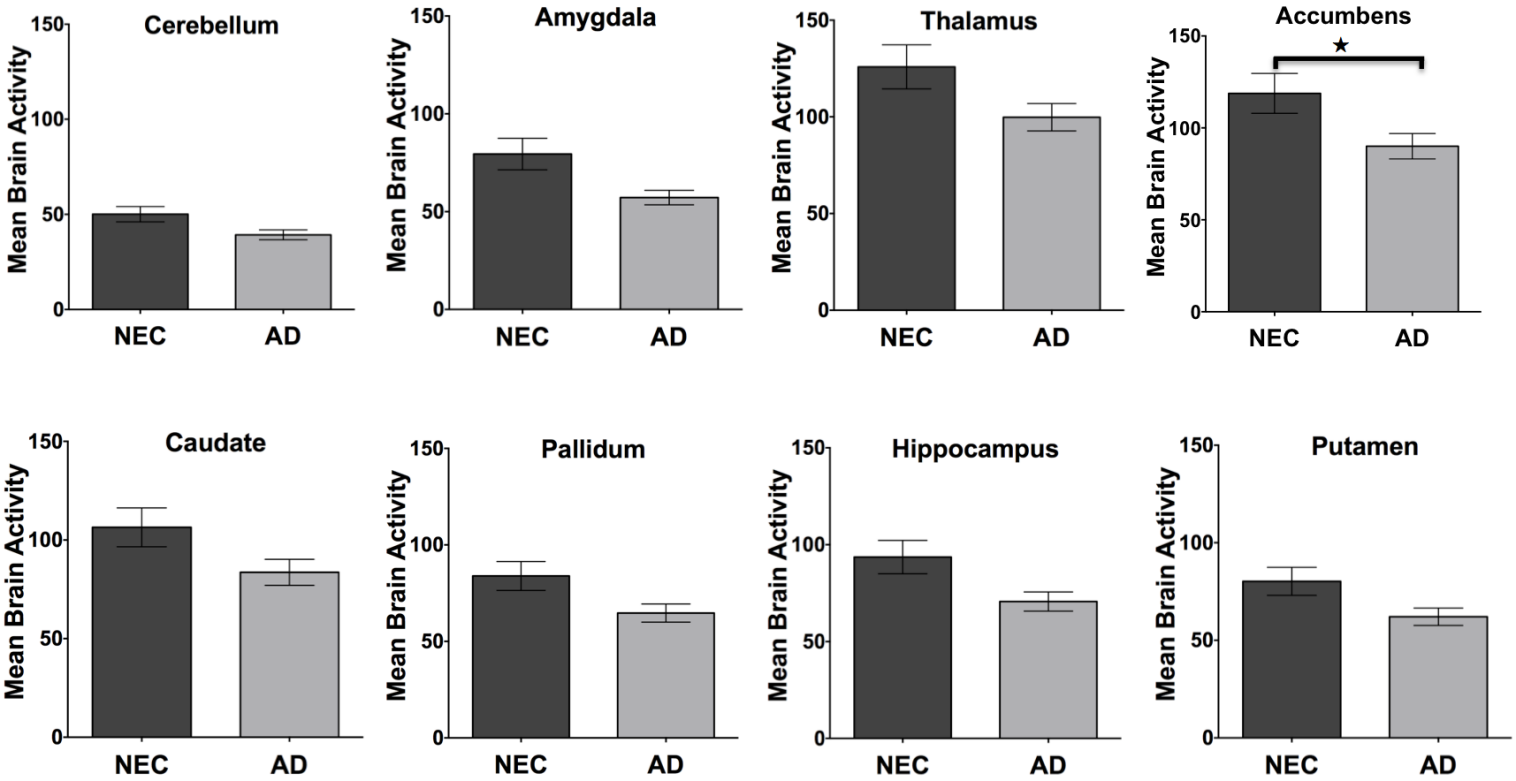
Regional brain activity. The average brain activity measured by RS-fMRI in NEC and Alzheimer disease subjects in the cerebellum, amygdala, thalamus, accumbens, caudate, pallidum, hippocampus, and putamen. The error bars represent the standard error of the mean and asterisks show significant differences between groups (adjusted *p* value, Bonferroni).

**Fig 5:**
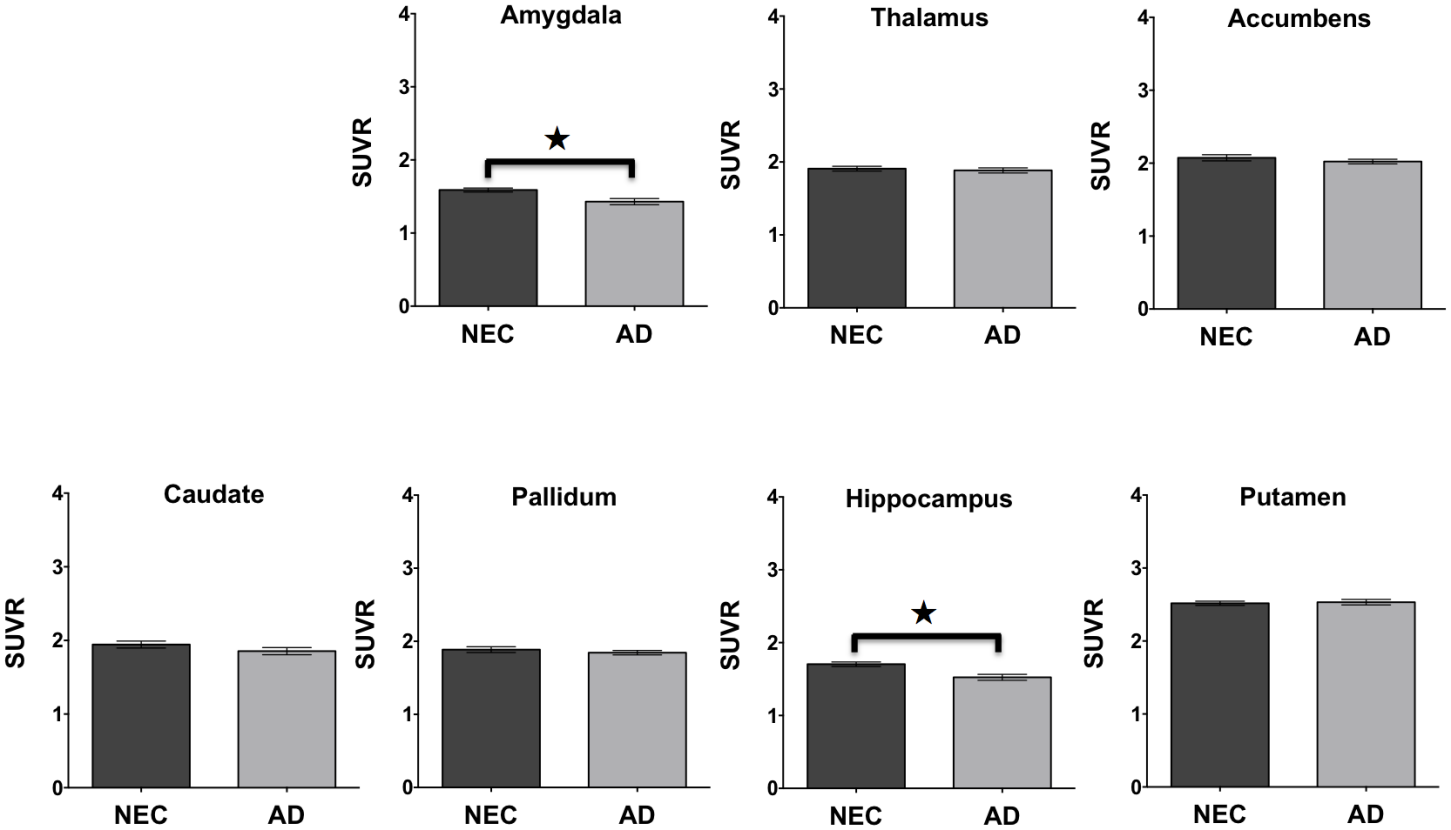
Regional glucose metabolism. The average glucose metabolism measured by FDG-PET SUVR in NEC and Alzheimer disease subjects in the amygdala, thalamus, accumbens, caudate, pallidum, hippocampus, and putamen. The error bars represent the standard error of the mean and asterisks show significant differences between groups (adjusted *p* value, Bonferroni).

The average brain activity measured from RS-fMRI (Fig 4) is shown for the cerebellum, amygdala, thalamus, accumbens, caudate, pallidum, hippocampus, and putamen regions for the NEC and Alzheimer disease groups. Similarly, the average relative rate of glucose metabolism (Fig 5) is provided for the same regions measured by FDG-PET for the NEC and Alzheimer disease groups. A one-way ANOVA indicated that there was a significant difference between the two groups in RS-fMRI brain activity (*p*<0.0001) and FDG-PET measured glucose metabolism (*p*<0.0001). In follow-up comparisons, the difference in brain activity measured by RS-fMRI in both sides of the accumbens (adjusted *p* = 0.04) and differences in relative glucose metabolism measured using FDG-PET in the amygdala (adjusted *p* = 0.02), and hippocampus (adjusted *p* = 0.006) remained significant following Bonferroni correction. A one-way ANOVA indicated that there was also a significant difference between these groups in the Tau, P-Tau and Aβ_1–42_ cerebral spinal fluid (CSF) levels (*p<*0.0001). Follow-up comparisons showed that there were significant differences in both Tau (adjusted *p*<0.0001), and Aβ_1–42_ (adjusted *p* = 0.006) following Bonferroni correction for multiple comparisons. As expected, a one-way ANOVA also showed a significant difference in normalized brain volumes between the NEC and Alzheimer disease groups (*p*<0.0001). Follow-up comparisons showed significant differences in normalized volume measured using MRI in the hippocampus (*p* = 0.01) between the two groups.

### Association between neuronal activity and glucose metabolism

**Fig 6:**
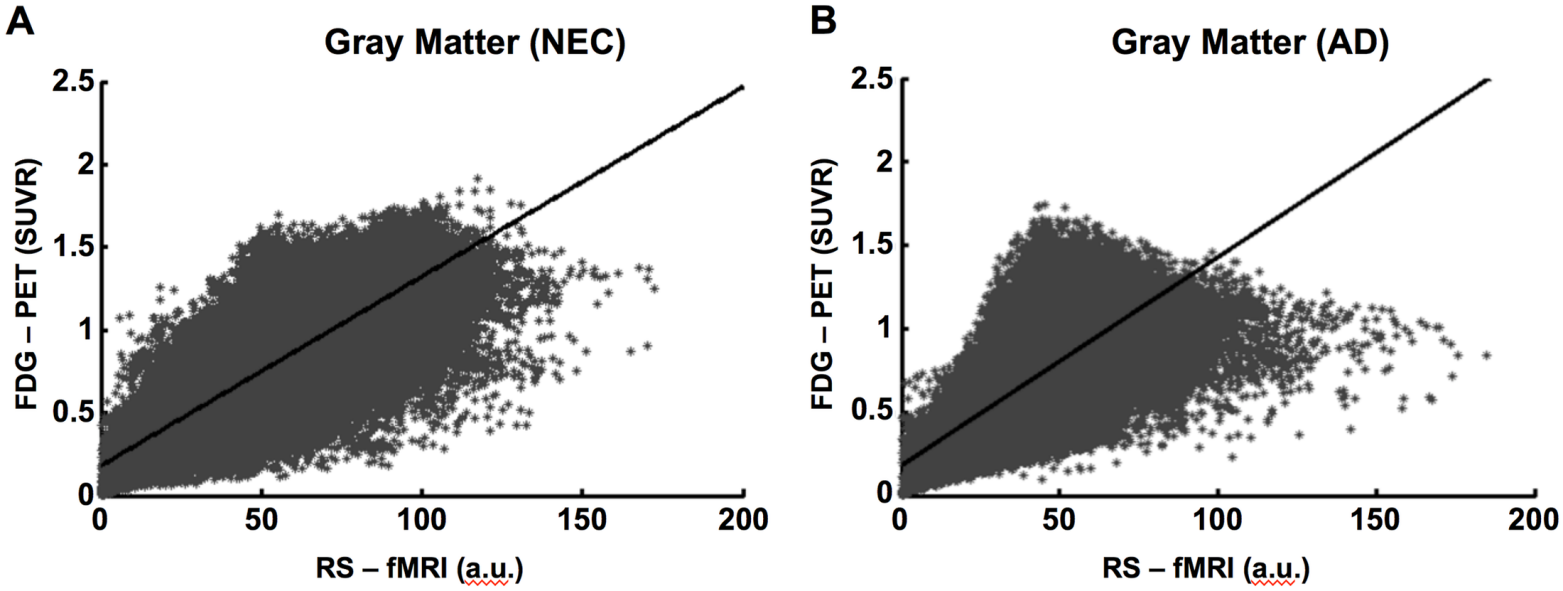
Relationship between glucose metabolism using FDG-PET and brain activity measured by RS-fMRI. Voxel by Voxel correlation of pixel intensity in gray matter (r = 0.81, *p*< 0.001) from a healthy elderly subject (A) and a patient with Alzheimer disease (B) (r = 0.77, *p*< 0.001) between brain activity using RS-fMRI and corrected glucose metabolism using FDG-PET SUVR.

A voxel by voxel correlation between gray matter RS-fMRI brain activity and the rate of glucose metabolism from the FDG-PET is shown in Fig 6 for one NEC (r = 0.81, *p*<0.0001) and subject with Alzheimer disease (r = 0.77, *p*<0.0001). Correlations were similar in all subjects (S1 and S2 Figs). The average r-value ± SD associated with the correlation between gray matter activity measured using RS-fMRI and the rate of glucose metabolism measured using FDG-PET was 0.77 ± 0.04 for the NEC group and 0.73 ± 0.03 for the Alzheimer disease group. The average *y*-intercept ± SD for the NEC group was 0.23 ± 0.03 and for the Alzheimer disease group was 0.23 ± 0.03.

### Comparison of neuronal components in NEC and Alzheimer disease

The average ± SD of the number of neuronal components was 6.4 ± 1.5 for the NEC group and 5.0 ± 1.2 for the Alzheimer disease group. Unpaired t-test (two-tailed) showed significantly fewer neuronal components in the Alzheimer disease group compared to the NEC group (*p* = 0.007). Pooling all subjects, the average number of neuronal components was positively correlated with MMSE score (r = 0.30, *p* = 0.002) (S3 Fig). The average ± SD of the RS-fMRI neuronal activity (Eq 2) summed across all neuronal components in whole brain was 54.8 ± 18.3 in the NEC group and 40.6 ± 10.4 in the Alzheimer disease group. Unpaired t-test (two-tailed) showed significantly lower neuronal activity in the Alzheimer disease group compared to the NEC group (*p* = 0.01) (S4 Fig).

### Association between neuronal activity and cognitive function

**Fig 7:**
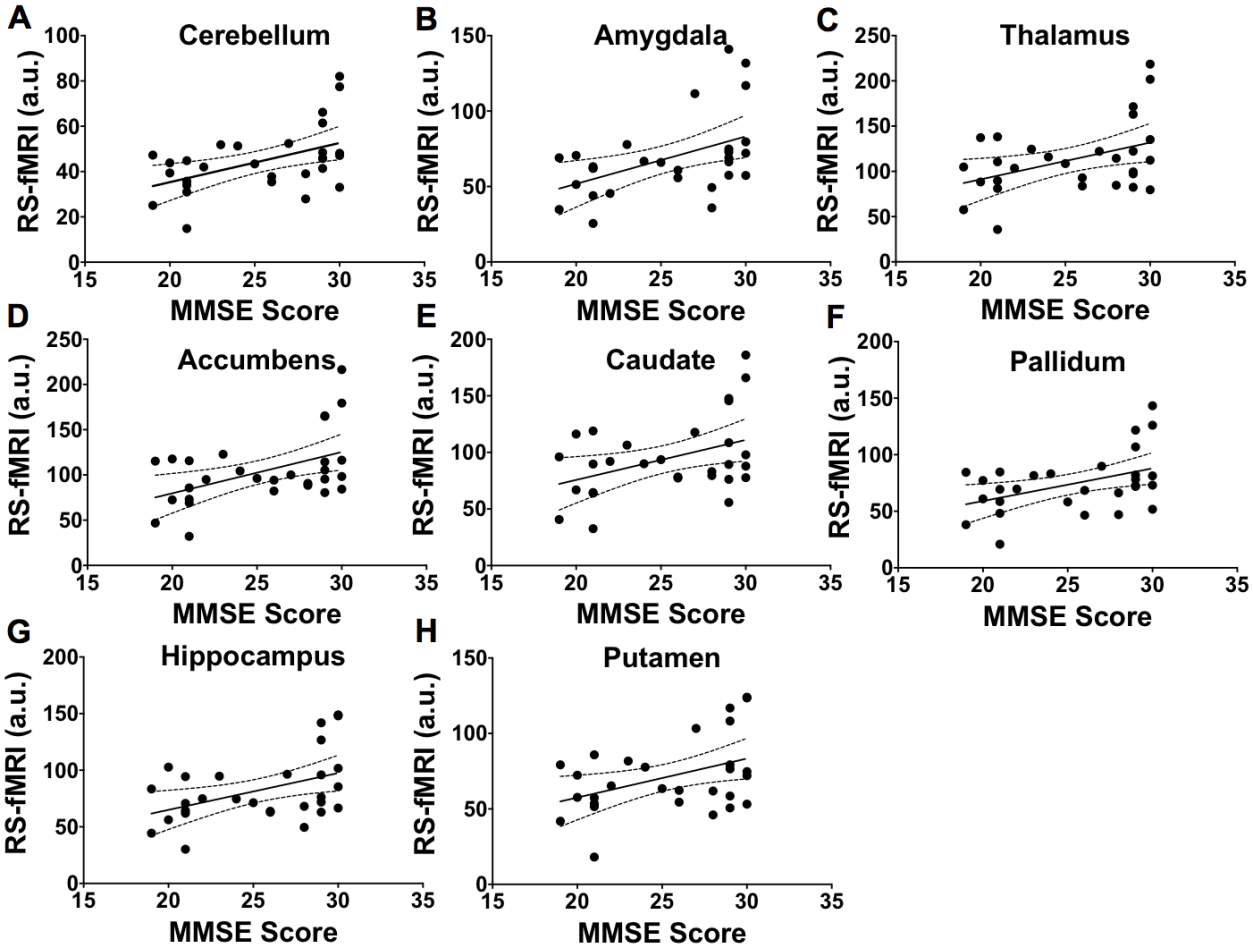
Relationship between mean brain activities measured using RS-fMRI and cognition function. The 95% confidence intervals for the regressions are shown as dotted lines. Association between MMSE score and brain activity in cerebellum (r = 0.50, *p* = 0.007, A), amygdala (r = 0.47, *p* = 0.009, B), thalamus (r = 0.42, *p* = 0.02, C), accumbens (r = 0.48, *p* = 0.008, D), caudate (r = 0.41, *p* = 0.02, E), pallidum (r = 0.44, *p* = 0.01, F), hippocampus (r = 0.44, *p* = 0.01, G), and putamen (r = 0.41, *p* = 0.02, H). These correlations were not significant following Bonferroni correction for multiple comparisons.

The relationship between RS-fMRI brain activity and clinical cognitive measurement is shown for several brain regions in Fig 7 when pooling all groups. However, the correlations between the MMSE score and brain activity in the cerebellum (r = 0.50, *p* = 0.007), amygdala (r = 0.47, *p* = 0.009), thalamus (r = 0.42, *p* = 0.02), accumbens (r = 0.48, *p* = 0.008), caudate (r = 0.41, *p* = 0.02), pallidum (r = 0.44, *p* = 0.01), hippocampus (r = 0.44, *p* = 0.01), and putamen (r = 0.41, *p* = 0.02) were not significant following Bonferroni correction.

### Association between neuronal activity and amyloid beta_1-42_

**Fig 8:**
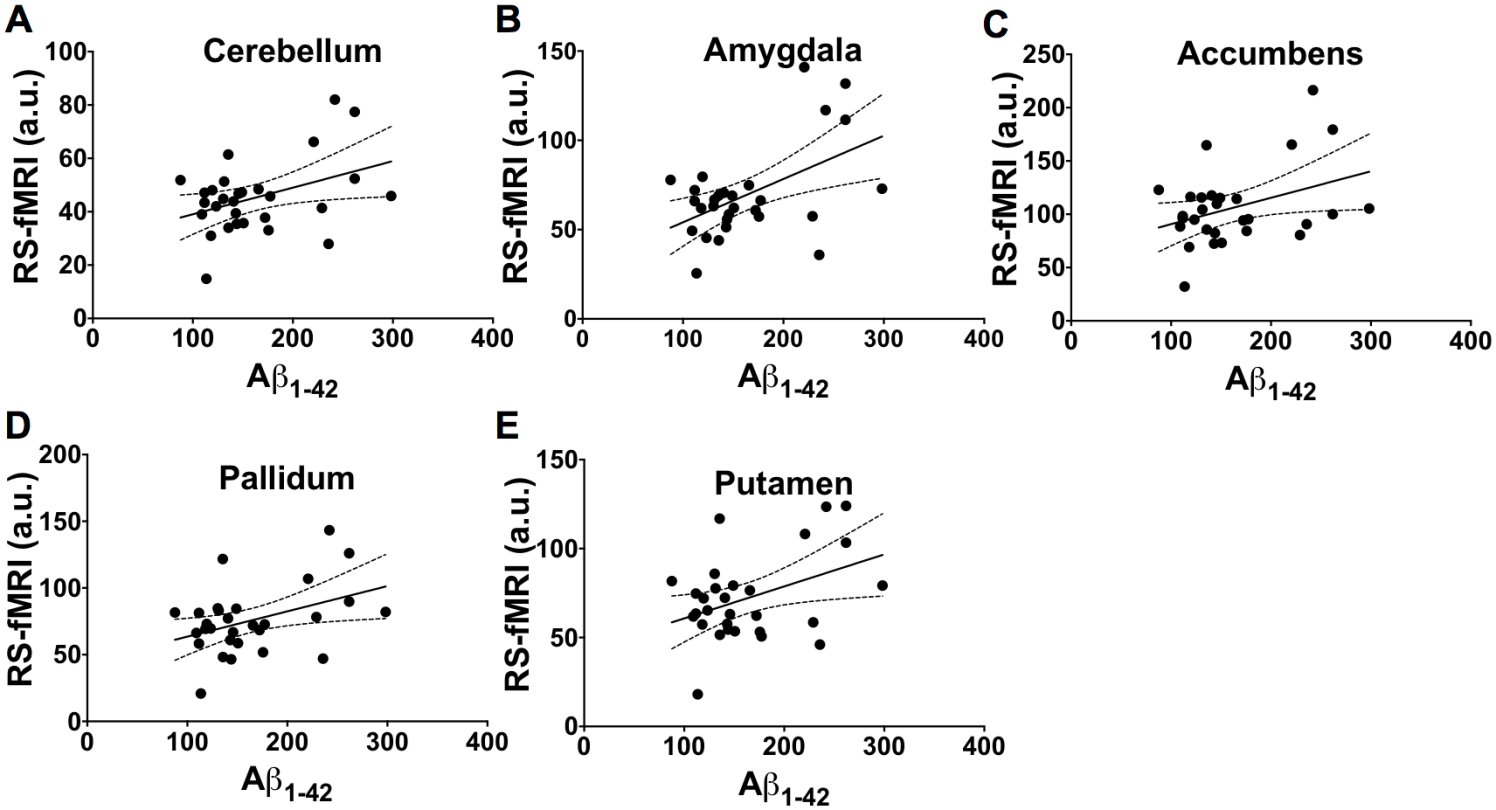
Relationship between mean brain activity measured using RS-fMRI and CSF derived Amyloid Beta_1-42_. The 95% confidence intervals for the regressions are shown as dotted lines. Association between the brain activity and Aβ_1–42_ in the cerebellum (r = 0.40, *p* = 0.03, A), amygdala (r = 0.51, *p* = 0.004, B), accumbens (r = 0.37, *p* = 0.04, C), pallidum (r = 0.41, *p* = 0.02, D), and putamen (r = 0.41, *p* = 0.02, E). These correlations were not significant following Bonferroni correction for multiple comparisons.

The relationship between RS-fMRI brain activity and Aβ_1–42_ CSF level is shown for several brain regions in Fig 8 for all subjects. However, the correlations between the RS-fMRI brain activity and Aβ_1–42_ in the cerebellum (r = 0.40, *p* = 0.03), amygdala (r = 0.51, *p* = 0.004), accumbens (r = 0.37, *p* = 0.04), pallidum (r = 0.41, *p* = 0.02), and putamen (r = 0.41, *p* = 0.02) were not significant following Bonferroni correction.

### Comparison of classification accuracy

The classification accuracy of subjects using brain activity measured by RS-fMRI and relative glucose metabolism rate measured using FDG-PET is provided in Table 2. The greatest classification accuracy, sensitivity, and specificity were obtained when using FDG-PET measurement from the hippocampus. The classification model that used glucose metabolism in the hippocampus measured by FDG-PET achieved 85% average accuracy, 84% sensitivity and 85% specificity (Table 2). The models that used brain activity in the hippocampus or accumbens resulted in a lower accuracy, sensitivity, and specificity (Table 2). However, the model that combined normalized hippocampal volume and neuronal activity of RS-fMRI from the hippocampus achieved 80% average accuracy, 72% sensitivity and 89% specificity, providing a similar accuracy and specificity to that observed with FDG-PET glucose metabolism in the hippocampus.

**Table 2:** Classification results for RS-fMRI brain activity, glucose metabolism measured by FDG-PET SUVR, and normalized volume measured using MRI. The 95% confidence interval (CI) is provided in parentheses.

SVM_linear (leave-one-out-test)	Accuracy	Sensitivity	Specificity
RS-fMRI (Hippocampus)	0.75	0.70	0.78
		(0.68–0.75)	(0.70–0.82)
RS-fMRI (Accumbens)	0.56	0.42	0.68
		(0.42–0.57)	(0.58–0.70)
RS-fMRI (Hippocampus, and Amygdala)	0.65	0.57	0.71
		(0.40–0.60)	(0.69–0.80)
FDG-PET (Hippocampus)	0.85	0.84	0.85
		(0.77–0.85)	(0.75–0.85)
FDG-PET (Hippocampus, and Amygdala)	0.76	0.84	0.68
		(0.78–0.84)	(0.62–0.75)
MRI (Hippocampus)	0.67	0.64	0.70
		(0.60–0.68)	(0.68–0.74)
RS-fMRI (Accumbens) + MRI (Hippocampus)	0.70	0.63	0.73
		(0.60–0.75)	(0.73–0.80)
RS-fMRI (Hippocampus) + MRI (Hippocampus)	0.80	0.72	0.89
		(0.65–0.76)	(0.83–0.90)

SVM = support vector machine, FDG-PET = ^18^fludeoxyglucose-PET, RS-fMRI = resting state functional MRI.

## Discussion

In this study we define a new metric related to neuronal activity based on the square root of the temporal standard deviation of the RS-fMRI signal summed across all *neuronal components* within a pixel. As a proof of principle, this new RS-fMRI based metric was used to compare brain activity in normal elderly subjects and patients with mild Alzheimer disease. The brain activity measurement is dependent on both the number of neuronal components and the temporal fluctuation associated with each component. Overall lower brain activity was observed in the mild Alzheimer disease group, particularly within the accumbens. A similar comparison in the same subjects using FDG-PET also showed overall group differences indicating lower glucose metabolism in the amygdala, and hippocampus. In a pixel-by-pixel analysis, brain activity derived from RS-fMRI was found to be strongly correlated with glucose metabolism measured by FDG-PET in gray matter.

The FDG-PET results from the current study are consistent with previous FDG-PET studies that have shown reduced glucose metabolism in subjects with Alzheimer disease [28, 45]. Reduced glucose metabolism has also been observed in the posterior cingulate, temporal, parietal lobes and later the frontal lobe [28, 46]. Mosconi *et*. *al*. [47] showed that glucose metabolism in the hippocampus and the entorhinal cortex is also reduced in the preclinical stage of Alzheimer disease. In addition, Jagust *et*. *al*. [48] has demonstrated that medial temporal and parietal glucose metabolism predicts cognitive decline. Glucose consumption measured by FDG-PET is also linearly associated with neuronal activity [49]. Therefore glucose consumption is considered an important indicator of presymptomatic Alzheimer disease [50].

A few RS-fMRI studies have also examined functional *network* activity in healthy subjects compared to people with Alzheimer disease. Seed based analyses [17, 18] have shown decreased functional connectivity (FC) in the medial temporal cortex, prefrontal cortex, precuneus, posterior cingulate, hippocampus, and thalamus in people with Alzheimer disease. Similar studies using ICA methods demonstrated reduced FC in precuneus, posterior cingulate, and parietal lobe in people with amnestic mild cognitive impairment (aMCI) [51] while Zhou *et*. *al*. [52] showed decreased FC in the default mode network including the hippocampus and the medial temporal lobe in people with Alzheimer disease. In the current study we did *not* examine functional connectivity, but instead used the RS-fMRI neuronal signal fluctuation to define a new indicator of brain activity. When examining activity in specific brain regions, the newly defined measurement of neuronal activity showed trends toward correlation with the MMSE score and Aβ_1–42_, but these associations did not remain significant following Bonferroni correction.

The new RS-fMRI metric related to brain activity showed regional differences between NEC and Alzheimer disease subjects that were different than those observed with FDG-PET. Previous studies have found that cerebral blood flow is closely coupled with brain metabolism [53, 54], and that both are decreased in people with Alzheimer’s disease [55–59]. Previous studies have also shown that there is an association between the resting brain activity and resting brain metabolism [60]. The strong correlation observed between RS-fMRI measured gray matter activity and FDG-PET measured glucose consumption in the current study is consistent with the notion that glucose metabolism is tightly coupled to the RS-fMRI measured brain activity. This result is also consistent with a recent study by Aiello *et*. *al*. [61] who showed a voxel-wise relationship between functional connectivity maps from RS-fMRI and glucose uptake measured by simultaneous FDG-PET in healthy subjects. Another recent study by Nugent *et*. *al*. [62] demonstrated that the correlation between functional connectivity maps derived from RS-fMRI and glucose metabolism measured by FDG-PET was lower in temporal lobe epilepsy patients compared to control subjects.

The group differentiation accuracy achieved using the brain activity metric in the accumbens measured by RS-fMRI was lower than that found when using FDG-PET measured glucose metabolism in the hippocampus (0.85). However, when combining hippocampal volume with brain activity from RS-fMRI in the hippocampus, the classification accuracy increased to 0.80, which is comparable to the FDG-PET results and significantly greater than using hippocampal volume alone. The specificity achieved when using the combined MRI-based measurements (0.89) was also greater than that achieved by FDG-PET and hippocampal volume alone. These results suggest that the combination of these MRI-based features could help to discriminate between a group of healthy elderly subjects and people with AD.

This proof of concept study demonstrates that decreased resting-state brain activity is associated with decreased brain glucose metabolism in mild Alzheimer disease. Furthermore, we demonstrated group classification based on a first-order textural feature (standard deviation) of the RS-fMRI neuronal signal. There are several limitations of the current study that should be considered. First, the sample size was limited by the availability of subjects with both ten-minute RS-fMRI data and FDG-PET. Regardless, the new metric was still able to differentiate between NEC and mild Alzheimer disease groups. Another limitation of this study is that we did not perform gray atrophy correction for either the RS-fMRI or FDG-PET signal changes. Therefore gray matter volume reduction in the hippocampus may partly explain the reduced RS-fMRI and FDG-PET measured brain activity. However, Mosconi *et*. *al*. [63] showed FDG-PET measured hypometabolism in the hippocampus despite atrophy correction in hippocampus. Gray matter atrophy measurements in the current study did show significant differences in the hippocampus between the two groups. However a classification model that included both the hippocampal volume and RS-fMRI brain activity showed an improved result to classification using either metric alone. Therefore, including this straightforward measurement of neuronal activity by RS-fMRI with existing markers of neurodegeneration may increase the reliability of detecting Alzheimer’s disease. Future studies will evaluate the potential to identify people with mild cognitive impairment that progress to AD and whether this metric shows improvement following treatment.

## Supporting information

S1 Fig The association between RS-fMRI and FDG-PET SUVR in the healthy elderly group. The scatter plots represent the voxel by voxel correlation of pixel intensity in the gray matter of healthy elderly subjects between brain activity using RS-fMRI and corrected glucose metabolism using FDG-PET SUVR. Each graph represents the results from a different individual.
(TIF)

S2 Fig The association between RS-fMRI and FDG-PET SUVR in the Alzheimer disease group. The scatter plots represent the voxel by voxel correlation of pixel intensity in the gray matter of Alzheimer disease subjects between brain activity using RS-fMRI and corrected glucose metabolism using FDG-PET SUVR. Each graph represents the results from a different individual.
(TIF)

S3 Fig The relationship between the number of neuronal components and cognition function measured by the MMSE score. The 95% confidence intervals for the regressions are shown as dotted lines. A significant correlation was found between MMSE score and the number of neuronal components (r = 0.30, *p* = 0.002).
(TIF)

S4 Fig Component specific brain activity maps in one healthy elderly subject and one subject with Alzheimer disease. Each image represents the square root of the standard deviation of the magnitude of the BOLD signal fluctuation. In this example, data from each identified neuronal component is provided in a different row. Eight neuronal components were identified in the healthy subject while only six neuronal components were identified in the subject with AD.
(TIF)

S1 Table Healthy elderly control subjects. (DOCX)

S2 Table People with Alzheimer’s disease. (DOCX)
