# Supplementary figures and images for: Spontaneous low frequency BOLD signal variations from resting-state fMRI are decreased in Alzheimer disease
Source: PLoS One. 2017 Jun 5;12(6):e0178529. doi: 10.1371/journal.pone.0178529 (PMC5459336; doi:10.1371/journal.pone.0178529)

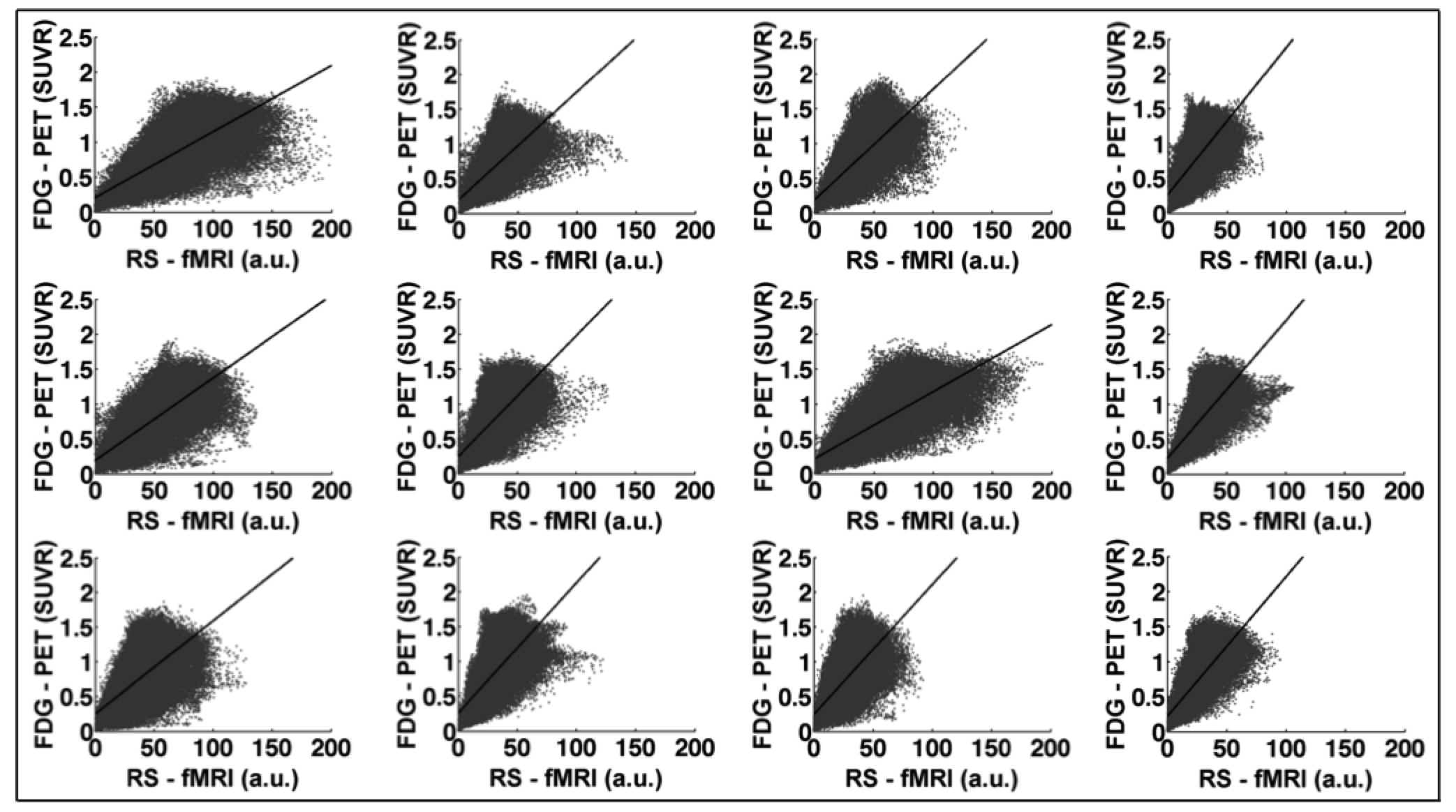

Supplement: S1 Fig — The scatter plots represent the voxel by voxel correlation of pixel intensity in the gray matter of healthy elderly subjects between brain activity using RS-fMRI and corrected glucose metabolism using FDG-PET SUVR. Each graph represents the results from a different individual. (TIF) [file pone.0178529.s001.tif]

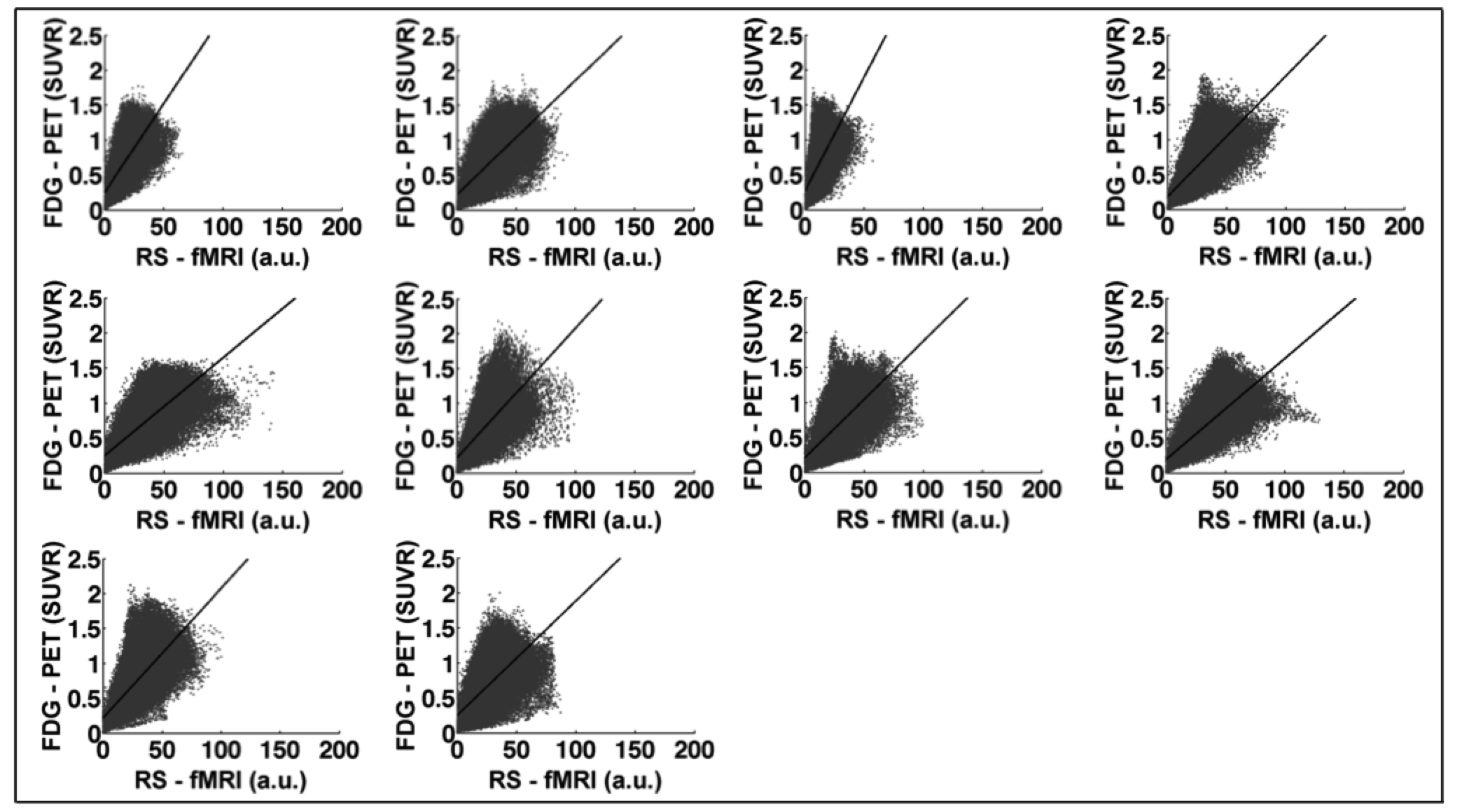

Supplement: S2 Fig — The scatter plots represent the voxel by voxel correlation of pixel intensity in the gray matter of Alzheimer disease subjects between brain activity using RS-fMRI and corrected glucose metabolism using FDG-PET SUVR. Each graph represents the results from a different individual. (TIF) [file pone.0178529.s002.tif]

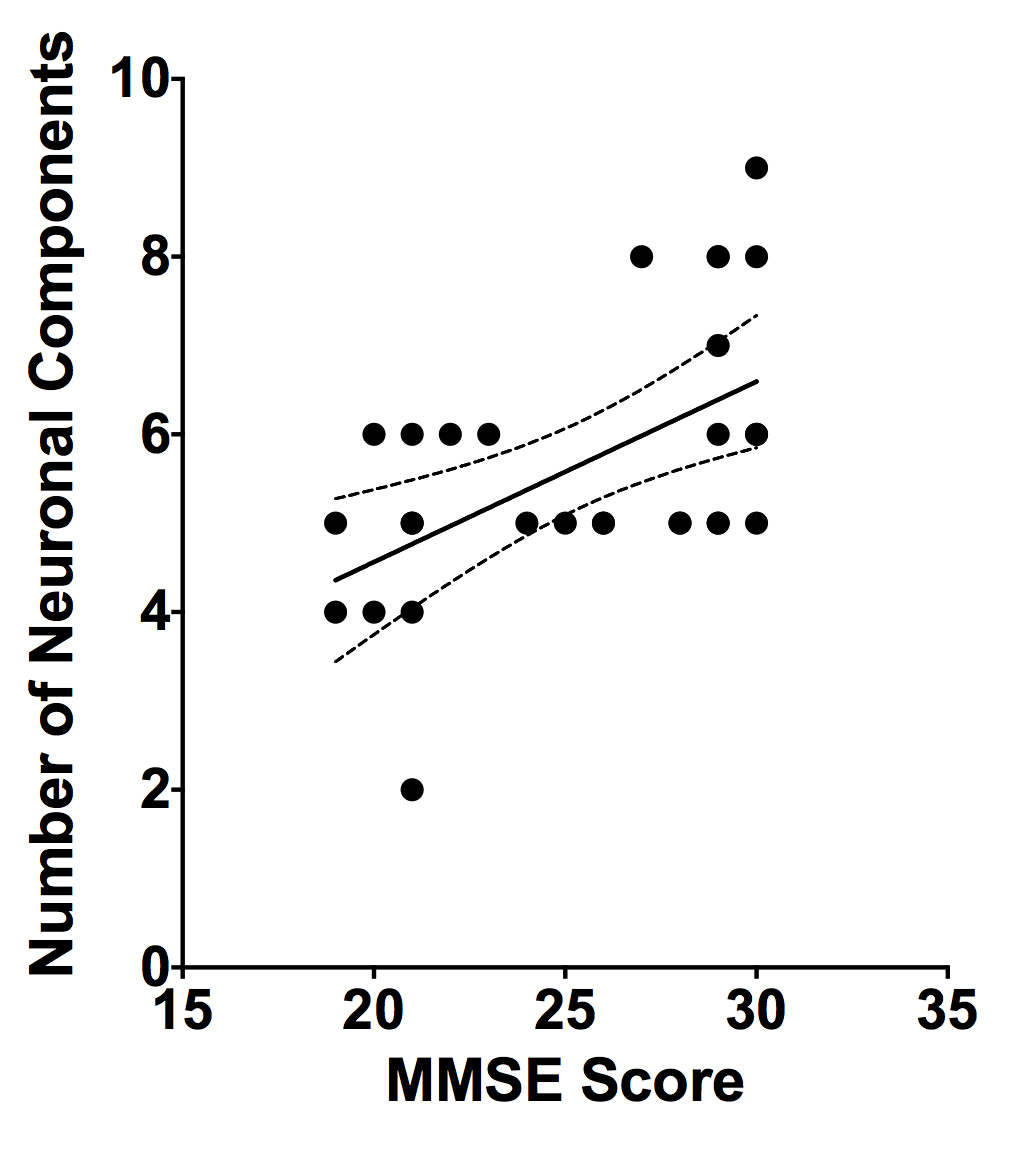

Supplement: S3 Fig — The 95% confidence intervals for the regressions are shown as dotted lines. A significant correlation was found between MMSE score and the number of neuronal components (r = 0.30, p = 0.002). (TIF) [file pone.0178529.s003.tif]

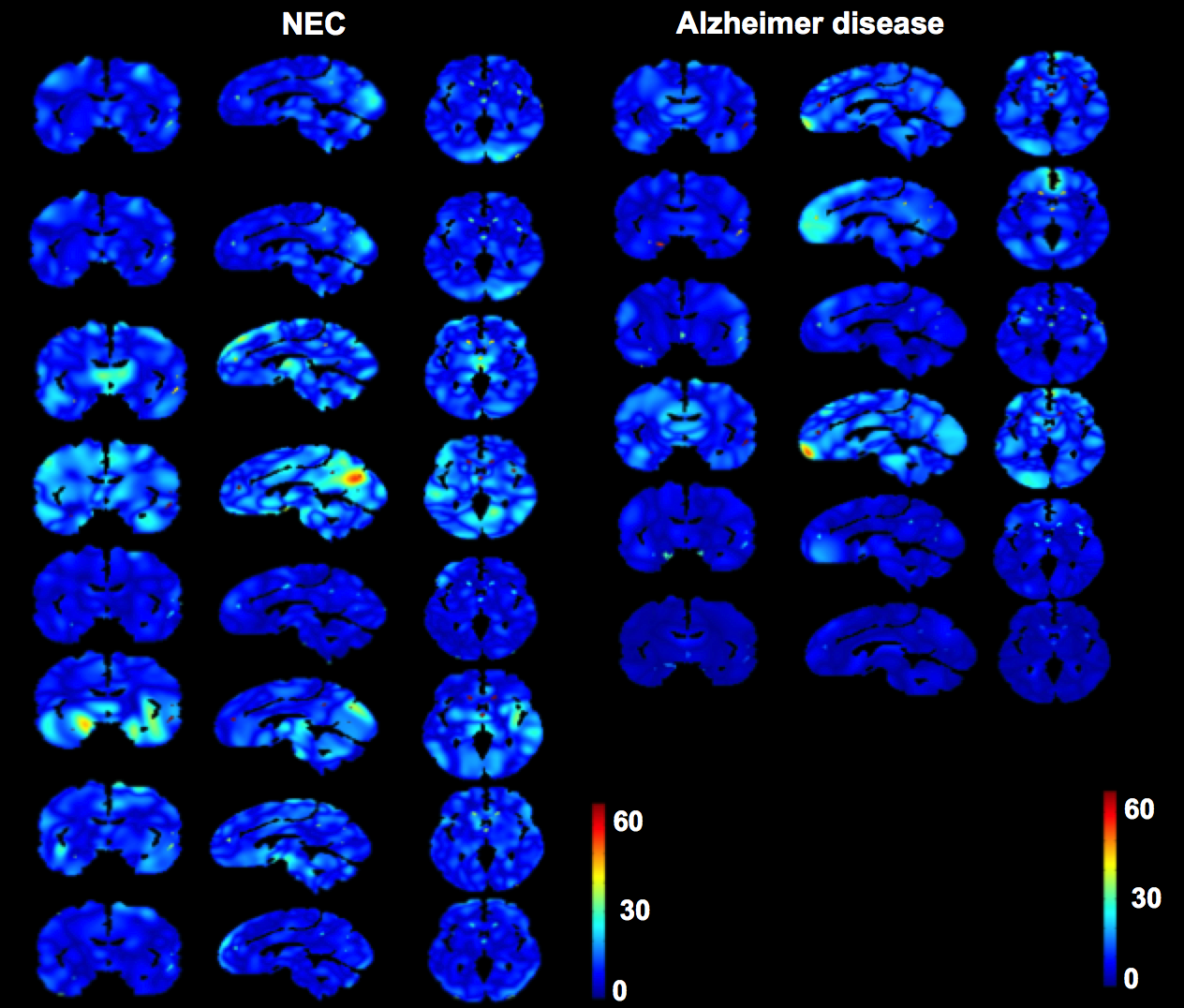

Supplement: S4 Fig — Each image represents the square root of the standard deviation of the magnitude of the BOLD signal fluctuation. In this example, data from each identified neuronal component is provided in a different row. Eight neuronal components were identified in the healthy subject while only six neuronal components were identified in the subject with AD. (TIF) [file pone.0178529.s004.tif]
