# Supplementary material for: Spontaneous low frequency BOLD signal variations from resting-state fMRI are decreased in Alzheimer disease
Source: PLoS One. 2017 Jun 5;12(6):e0178529. doi: 10.1371/journal.pone.0178529 (PMC5459336; doi:10.1371/journal.pone.0178529)
Supplement: S1 Table — (DOCX) [file pone.0178529.s005.docx]

| **ADNI Subject ID** | **Scan Date** |
| --- | --- |
| 002_S_0413 | 2011-06-17 |
| 002_S_4213 | 2011-09-03 |
| 002_S_4262 | 2011-10-05 |
| 002_S_4270 | 2011-10-11 |
| 010_S_4345 | 2012-02-01 |
| 010_S_4442 | 2012-02-10 |
| 012_S_4643 | 2012-04-16 |
| 013_S_4616 | 2012-04-23 |
| 013_S_4580 | 2012-04-11 |
| 018_S_4349 | 2012-07-10 |
| 019_S_4835 | 2012-07-11 |
| 031_S_4218 | 2011-09-15 |
| 053_S_4578 | 2012-03-17 |
| 100_S_4469 | 2012-03-09 |
| 136_S_4269 | 2011-11-01 |
