# Supplementary material for: Spontaneous low frequency BOLD signal variations from resting-state fMRI are decreased in Alzheimer disease
Source: PLoS One. 2017 Jun 5;12(6):e0178529. doi: 10.1371/journal.pone.0178529 (PMC5459336; doi:10.1371/journal.pone.0178529)
Supplement: S2 Table — (DOCX) [file pone.0178529.s006.docx]

| **ADNI Subject ID** | **Scan Date** |
| --- | --- |
| 002_S_5018 | 2012-11-12 |
| 013_S_5071 | 2013-02-15 |
| 018_S_4733 | 2012-08-30 |
| 018_S_4696 | 2012-08-03 |
| 019_S_4549 | 2012-04-24 |
| 019_S_4252 | 2011-09-29 |
| 019_S_4477 | 2012-01-26 |
| 019_S_5012 | 2012-11-05 |
| 019_S_5019 | 2012-11-08 |
| 130_S_4589 | 2012-03-21 |
| 130_S_4660 | 2012-04-23 |
| 130_S_4982 | 2012-10-23 |
| 130_S_4984 | 2012-10-25 |
| 130_S_4997 | 2012-11-20 |
| 130_S_5006 | 2012-11-30 |
